# On your marks, get SET(D1A): the race to protect stalled replication forks

**DOI:** 10.1080/23723556.2018.1511209

**Published:** 2018-10-11

**Authors:** Shabana Begum, Amalia Goula, Rachel Bayley, Martin R. Higgs

**Affiliations:** Lysine Methylation and DNA Damage Laboratory, Institute of Cancer and Genomic Sciences, University of Birmingham, Birmingham, UK

**Keywords:** Genome stability, SETD1A, FANCD2, replication stress, Chemoresistance, CHD4

## Abstract

We recently identified that methylation of lysine 4 of histone H3 (H3K4) by SETD1A (SET domain containing 1A) maintains genome stability by protecting newly-replicated DNA from degradation. Mechanistically, SETD1A-dependent histone methylation regulates nucleosome mobilisation by FANCD2 (FA complementation group D2), a crucial step in maintaining genome integrity with important implications in chemo-sensitivity.

Duplication of the cellular genome by DNA replication is a highly regulated process crucial for cellular and organismal homeostasis. Compromising this regulation leads to slowing or stalling of replication forks, known as replication stress, which if unresolved gives rise to genome instability.^1^ Signatures of replication stress-associated genetic damage are observed in pre-cancerous as well as tumoural cells. Therefore, since loss of genomic integrity is a well-recognised hallmark of cancer, DNA replication stress is likely a key driver of cancer development.

Given this, it is unsurprising that numerous factors have developed to counteract the negative consequences of unresolved replication stress, preserving genome stability and maintaining human health. One key event mediated by these proteins is the active reversal of paused/stalled replication forks;^2^ the formation of these reversed forks is vital in protecting genome integrity. Moreover, they must also be protected from uncontrolled degradation by cellular nucleases (reviewed in ref)^3^: if forks are unprotected and subsequently degraded, this also leads to severe genome instability. Reversed forks are protected through the actions of a number of factors including the tumour suppressor genes BRCA1 (BRCA1, DNA repair associated) and BRCA2 (BRCA2, DNA repair associated), as well as other components of the homologous recombination and Fanconi anaemia (FA) repair pathways. Crucially, loss of fork protection has potential clinical implications during cancer therapy, since the ability of tumour cells to acquire drug resistance is intimately linked to their ability to protect replication forks from degradation,^4^(Figure 1A-B). However, despite the clinical importance of fork protection, we do not fully understand how reversed forks are marked to elicit protection, and in particular how post-translational chromatin modifications may play a role. A recent publication from our group in Molecular Cell has shed further light on the mechanisms by which cells label stalled replication forks for protection.^5^ We identified that protection of replication forks under conditions of replication stress requires SETD1A (SET domain containing 1A), a member of the KMT2 family of lysine methyltransferases, well known for their ability to catalyse methylation of lysine 4 of histone H3 (H3K4). We showed that SETD1A depletion exposes reversed replication forks to nucleolytic degradation upon replication stress, increasing genome instability and hyper-sensitising cells to agents that induce replication stress. Moreover, although both SETD1A and SETD1B (SET domain containing 1B, a closely related member of the KMT2 family) methylate H3K4, only the catalytic activity of SETD1A is required for fork protection. Mechanistically, by methylating H3K4 at/near reversed replication forks, SETD1A prevents the RAD51 recombinase from being destabilised away from these sites, safeguarding nascent DNA from uncontrolled resection by the helicase/nuclease DNA2.

**Figure 1.**
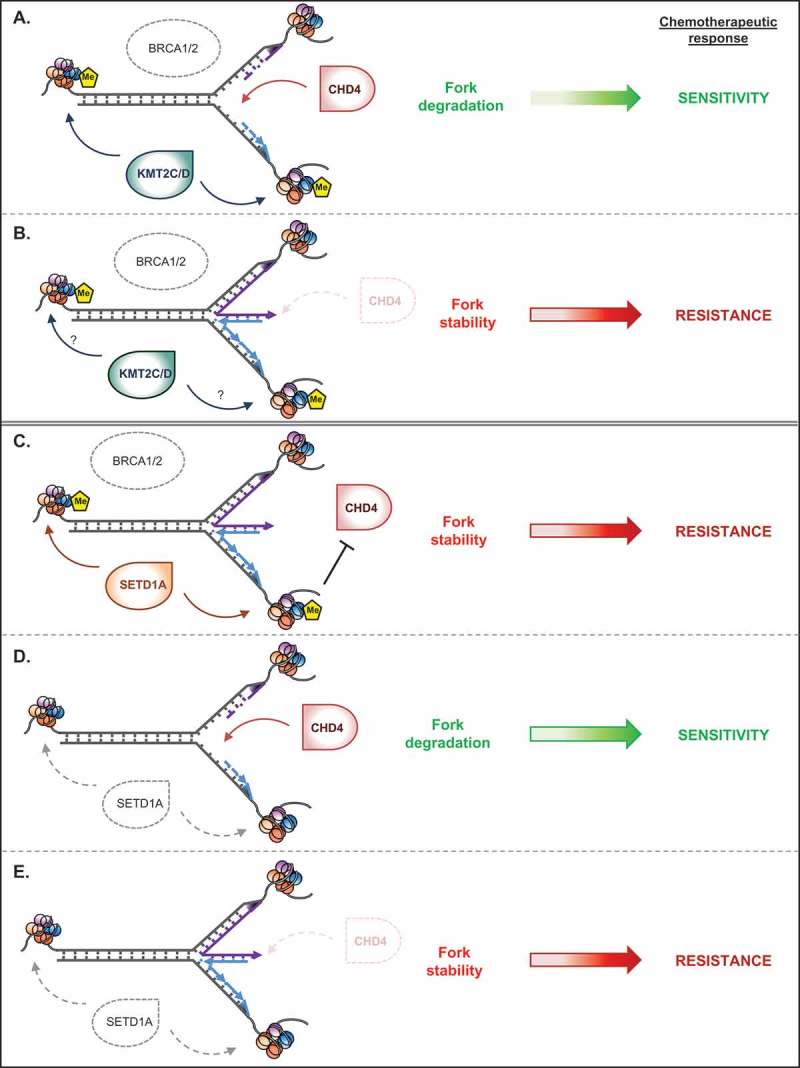
**Histone methylation, fork protection and chemo-resistance. A)** In the absence of BRCA1/BRCA2 tumour suppressors (dotted oval), fork degradation is driven by the helicase CHD4 and by KMT2C/KMT2D^4^. This fork degradation gives rise to the chemotherapeutic sensitivity of tumoural cells. Histone methylation on lysine 4 of histone H3 (H3K4 Me; yellow pentagons) by KMT2C/KMT2D seems to have no effect on CHD4 activity in this context. **B)** Loss of CHD4 in *BRCA*-deficient cells restores fork stability, giving rise to resistance to chemotheurapeutic agents such as mitomycin C. **C)** In contrast, our paper^5^ demonstrates that SETD1A-mediated histone methylation protects forks, even in the absence of BRCA1/BRCA2. This would render tumoural cells less sensitive to replication-stress inducing agents. **D)** Upon loss of SETD1A (dotted teardrop), loss of H3K4 methylation allows CHD4 to drive fork degradation, leading to fork instability and chemo-sensitivity. **E)** As in *BRCA* -deficient cells, loss of CHD4 restores fork stability but leads to chemotherapeutic resistance. Thus, restoration of fork protection through disparate mechanisms in different genetic contexts accounts for the development of chemo-resistance in tumoural cells.

Importantly, this SETD1A-dependent protection is mediated by controlling access/activity of chromatin remodelling factors: firstly, SETD1A-dependent H3K4 methylation limits fork degradation by preventing access of Chromodomain helicase DNA binding protein 4 (CHD4; a nucleosome remodelling factor and component of the NuRD complex) to reversed forks (Figure 1C). Although it is unclear how CHD4 promotes fork degradation, we hypothesise that it may promote chromatin ‘shuffling’ and therefore allow access of nucleases to newly synthesized DNA. Secondly, we showed that SETD1A and H3K4 methylation functions together with the DNA-damage dependent histone chaperone FANCD2^6^ (FA complementation group D2) to enhance histone mobility at stalled forks, further protecting these structures from degradation by helping to stabilise the RAD51 recombinase. Thus, our findings suggest that epigenetic histone modifications catalysed by SETD1A are required to tightly control the access and/or function of histone remodelling factors at stalled replication forks, preventing nucleolytic degradation of nascent DNA.

It is clear from a number of studies, including ours, that the KMT2 family of H3K4 KMTs play crucial roles in controlling the recruitment and/or activity of factors that regulate fork stability. Interestingly, other KMTs (such as EZH2^7^ (Enhancer of zeste 2 polycomb repressive complex 2 subunit)) also play a role in fork protection, further underlining the importance of epigenetic modifications in this process. However, perhaps counter-intuitively, different KMT2 enzymes elicit different responses within similar genetic backgrounds, despite the fact that they all target H3K4 for methylation. This is illustrated by studies demonstrating that, in contrast to our findings, KMT2C & KMT2D (Lysine methyltransferase 2C & D; also known as MLL3 and MLL2 respectively) actually promote nascent strand degradation in the presence of impaired fork protection (e.g. *BRCA2*-deficient tumour cells)^4^. In contrast, it is clear from our findings^5^ that SETD1A functions constitutively to protect forks, regardless of whether or not fork stability has already been compromised, and that it regulates fork stability via numerous mechanisms. This discrepancy is in agreement with murine studies suggesting that there is little functional redundancy between the six KMT2 family members. Therefore, identifying how each KMT functions in different genetic backgrounds to protect forks is fundamental to further understanding acquired chemoresistance.

Despite the differing roles of BRCA1, BRCA2 and SETD1A in mediating fork protection, and the contrasts between SETD1A, SETD1B, KMT2C and KMT2D, it is also clear that different fork ‘protection factors’ can also prevent DNA degradation via common mechanisms. Indeed, both BRCA1/BRCA2 and SETD1A suppress CHD4-mediated fork degradation^4,5^ (Figure 1). Moreover, our findings have important clinical consequences, since loss of CHD4 in primary and *BRCA*-deficient tumour cells confers resistance to treatment with poly ADP ribose polymerase (PARP) inhibitors and cisplatin.^8^ Thus, CHD4 represents a common mechanism that drives fork degradation in the absence of protective factors, perhaps by promoting fork reversal or by regulating local chromatin environments, and this in turn leads to chemo-sensitivity. Loss of CHD4 in these genetic backgrounds would therefore engender chemo-resistance in what would otherwise be sensitive tumour cells (Figure 1B and E). Therefore, identifying how the cell protects nascent DNA is a crucial step towards defining the mechanisms by which tumour cells acquire resistance to commonly used chemotherapeutic agents.

Much more work clearly remains in elucidating how different members of the same KMT family elicit such markedly different effects within diverse genetic backgrounds. Moreover, future studies on the regulation of lysine methylation, and the identification of other KMTs that play a role in this process, would contribute substantially towards our understanding of fork stability. This in turn could open new and exciting avenues towards the development of more efficient chemotherapeutic agents or strategies.
